# Supplementary material for: Effects of Lactiplantibacillus plantarum GUANKE on Diphenoxylate-Induced Slow Transit Constipation and Gut Microbiota in Mice
Source: Nutrients. 2023 Aug 26;15(17):3741. doi: 10.3390/nu15173741 (PMC10490327; doi:10.3390/nu15173741)
Supplement: Supplementary file 1 [file nutrients-15-03741-s001.zip › nutrients-2563190-supplementary.pdf]

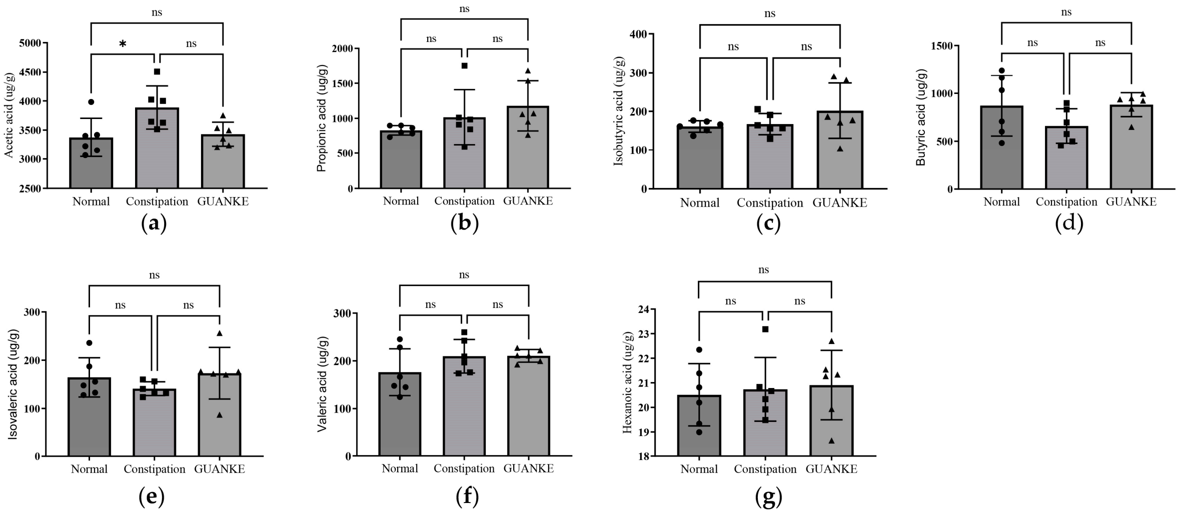

Figure S1. Effects of *L. plantarum* GUANKE on SCFA profiles of intestinal factors associated with constipation in mice. (**a**) Acetic acid; (**b**) propionic acid; (**c**) isovaleric acid; (**d**) butyric acid; (**e**) isobutyric acid; (f) valeric acid; (**g**) hexanoic acid. Statistical analysis was performed using one-way ANOVA followed by LSD (least significant difference) for multiple comparisons of groups. * $p < 0.05$, ns = no significant difference ($p > 0.05$).
